# Supplementary material for: Learning curve and surgical outcome of robotic assisted colorectal surgery with ERAS program
Source: Sci Rep. 2022 Nov 29;12:20566. doi: 10.1038/s41598-022-24665-w (PMC9709162; doi:10.1038/s41598-022-24665-w)
Supplement: Supplementary file 2 — Supplementary Information 2. [file 41598_2022_24665_MOESM2_ESM.zip › RAL Raw data-Ñ[▒K/078/OP_ñw╝╨░O▒Kñσ.pdf]

正式摘要(電子簽章：000000215651588)

W95 / W95

臺中榮民總醫院

Operation Note

[redacted]

- 手術日期：2021/04/23
- 手術醫師：VS 蔣鋒帆，VS 黃伲霖，CR 林陳彥，R 陳勇先
- Anesthesia : GE
- Aseptic agents : Betadine Aqua , Zephnan

Diagnosis

- Pre-operative diagnosis : Constipation – functional, change
- Post-operative diagnosis : s/p robotic subtotal colectomy

Surgical Indication

- For functional problem

Operation

- 1. Robotic (XI) assisted subtotal colectomy
  2. Intraoperative colonoscopy : (-).
  3. High ligation (-) : The root was not resected.
  4. Take down from the splenic flexure : (+)
  5. Take down from the hepatic flexure : (+)
  6. OpenGIA (75-1.5) X2 were applied to complete the side-to-end anastomosis
  7. Proximal cut end: 10 cm (Small bowel).
  8. No drainage tube was inserted
  9. ADHESION BARRIER FILM was placed below the central extended wound

Finding

- 1. No suspicious tumor was identified.
  2. The colon was redundant.
  3. No obvious ascites was found.

Procedure

- 1. General endotracheal anesthesia was induced and then the patient was placed in modified Trendelenburg lithotomy position.
  2. Preoperative antibiotics were given. A Foley catheter and nasogastric tube were placed.
  3. The abdomen was prepped and draped in standard sterile fashion.
  4. A small horizontal incision was made below the umbilicus and a 8 mm trocar was inserted into the peritoneal cavity by open method. Peumoperitoneum was established with CO2 inflation.
  5. Laparoscopy was inserted and then other two 8mm trocars were inserted over LLQ, RLQ of abdomen. Another 12mm trocars was inserted over LLQ area, and the other assist-port 5mm troca was inserted on LLQ. The operative findings were mentioned as above.
  6. Omentum was freed from the transverse colon and lesser sac was entered.
  7. The distal branch of middle colic artery were all further ligated via Hemo-clips.
  8. The dissection was performed carefully toward both hepatic flexure and splenic flexure.
  9. The colon was freed from its peritoneal attachments along the line of Toldt from the cecum to the hepatic flexure.
  10. The peritoneum overlying the mesentery was then scored with electrocautery and the ileocolic artery was identified, ligated with endoclips
  11. Hemostasis was checked in the operative field. Cecum, ascending colon and hepatic flexure were mobilized after the right colic artery and other small vessels were ligated.
  12. The sigmoid colon was held by grasper and the peritoneum overlying the mesentery was scored with electrocautery.

- 13. The inferior mesentery artery was identified, ligated above the left colic artery with robotic clips, and transected. IMV was ligated via endoclip and then also transected.
  14. Dissection was done above the Gerota's fascia and toward the peritoneal attachments.
  15. The colon was mobilized from retroperitoneum overlying the sacral promontory and into the pelvis.
  16. Distal descending colon and s-colon were freely mobilized after the mesoclon was all dissected.
  17. The trochar wound below the umbilicus was further vertically extended toward.
  18. Wound retractor (ALEXIS WOUND RETRACTOR) was then set up.
  19. The colon and the terminal ileum was then all retracted out for examination.
  20. The desired resection site was selected and the, the side to side anastomosis was completed via firing 2 staples of OpenGIA (75-1.5).
  21. Interrupted 3-0 silk Lambert sutures were used to re-inforcement at outer layer
  22. The trochar fascia wounds were all then closed with interrupted sutures via 1-O Vicryl.
  23. ADHESION BARRIER FILM was placed below the central extended abdominal wound.
  24. The extended fascia wound was closed with interrupted sutures via Num.1 Vicryl.
  25. Massive N/S was applied to irrigate the wound.
  26. The wound was further closed with 3-O Vicryl, and the skin was applied with SurgiSeal and covered with Medipore.
  27. The patient tolerated the procedure well and was brought to the POR in stable condition.
- Estimated blood loss: minimum
- 器械數量: 正確
- 紗布數量: 正確

Image

- PAINT
  - abdomen

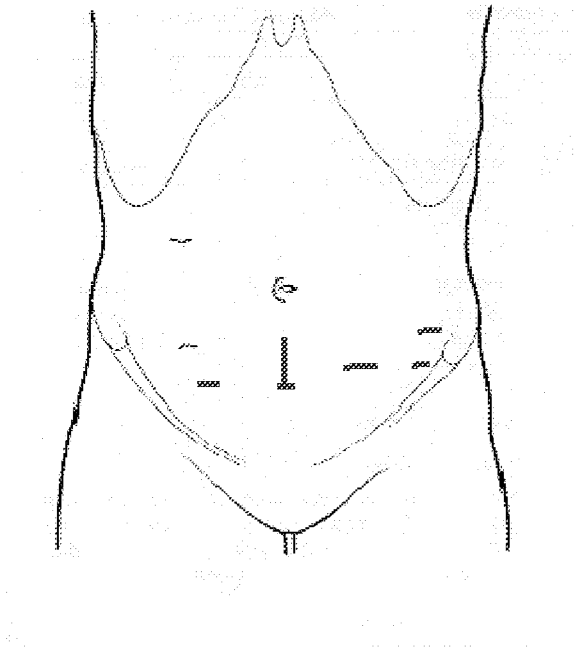

  - colon & rectum

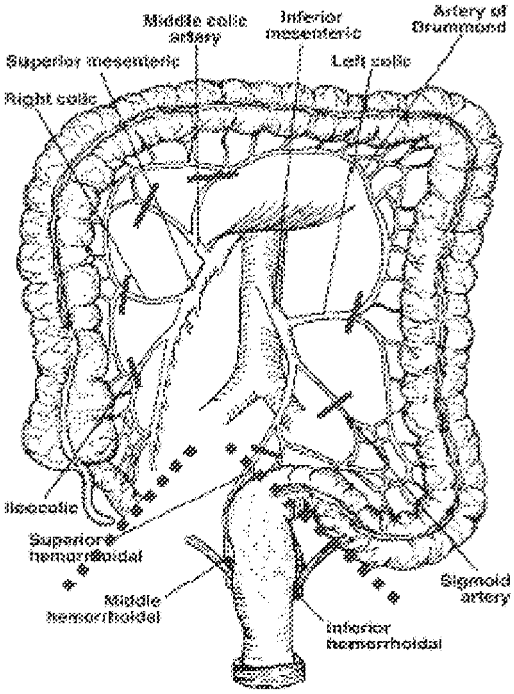

- The resected specimen

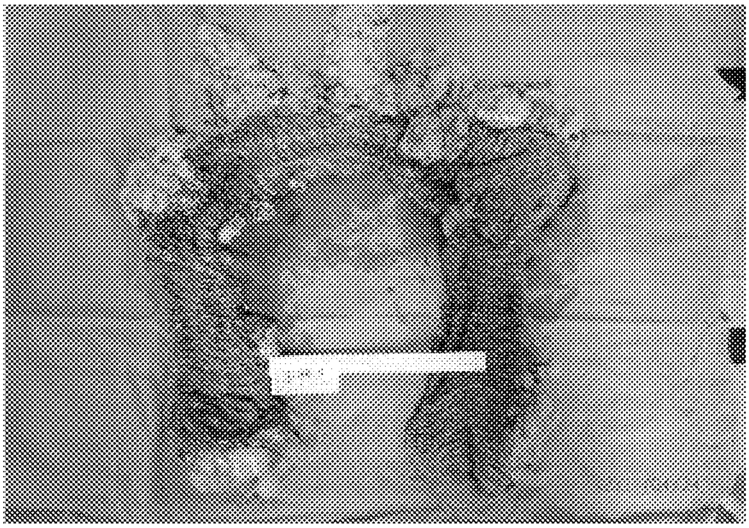

主任 2062F蔣鋒帆 / 醫師 2301H林陳彥

正式摘要(電子簽章：000000215651588)

W95 / W95

doctorRank:B
